# Solanesol Biosynthesis in Plants

**DOI:** 10.3390/molecules22040510

**Published:** 2017-03-23

**Authors:** Ning Yan, Yanhua Liu, Hongbo Zhang, Yongmei Du, Xinmin Liu, Zhongfeng Zhang

**Affiliations:** Tobacco Research Institute of Chinese Academy of Agricultural Sciences, Qingdao 266101, China; liuyanhua@caas.cn (Y.L.); zhanghongbo@caas.cn (H.Z.); duyongmei@caas.cn (Y.D.); liuxinmin@caas.cn (X.L.); zhangzhongfeng@caas.cn (Z.Z.)

**Keywords:** solanesol, function, biosynthetic pathway, key enzymes

## Abstract

Solanesol is a non-cyclic terpene alcohol composed of nine isoprene units that mainly accumulates in solanaceous plants. Solanesol plays an important role in the interactions between plants and environmental factors such as pathogen infections and moderate-to-high temperatures. Additionally, it is a key intermediate for the pharmaceutical synthesis of ubiquinone-based drugs such as coenzyme Q10 and vitamin K2, and anti-cancer agent synergizers such as *N*-solanesyl-*N*,*N*′-bis(3,4-dimethoxybenzyl) ethylenediamine (SDB). In plants, solanesol is formed by the 2-*C*-methyl-d-erythritol 4-phosphate (MEP) pathway within plastids. Solanesol’s biosynthetic pathway involves the generation of C5 precursors, followed by the generation of direct precursors, and then the biosynthesis and modification of terpenoids; the first two stages of this pathway are well understood. Based on the current understanding of solanesol biosynthesis, we here review the key enzymes involved, including 1-deoxy-d-xylulose 5-phosphate synthase (DXS), 1-deoxy-d-xylulose 5-phosphate reductoisomerase (DXR), isopentenyl diphosphate isomerase (IPI), geranyl geranyl diphosphate synthase (GGPPS), and solanesyl diphosphate synthase (SPS), as well as their biological functions. Notably, studies on microbial heterologous expression and overexpression of key enzymatic genes in tobacco solanesol biosynthesis are of significant importance for medical uses of tobacco.

## 1. Introduction

Solanesol is a non-cyclic terpene alcohol composed of nine isoprene units, and serves as a key intermediate in the synthesis of ubiquinone-based drugs and supplements such as coenzyme Q10, vitamin K2, and the anti-cancer agent synergizer *N*-solanesyl-*N*,*N*′-bis(3,4-dimethoxybenzyl) ethylenediamine (SDB) [1,2,3]. Solanesol was first isolated from tobacco (*Nicotiana tabacum*), and has been subsequently reported in other solanaceous plants, including potato (*Solanum tuberosum*), tomato (*Solanum lycopersicum*), eggplant (*Solanum melongena*), and pepper (*Capsicum annuum*) [3,4]. Tobacco has the highest solanesol content amongst all solanaceous plants. De novo synthesis of solanesol is extremely challenging because of its long carbon chains [5]; hence, solanesol production remains reliant on extraction from tobacco leaves [3,6]. The content of solanesol in plants is jointly determined by genetic and environmental factors; pathogen infection, moderately high temperature, moderate drought, moderate shade, long-wavelength/extended irradiation, rare-earth elements, and tobacco topping all increase the solanesol content of tobacco [1,3,7]. Therefore, deeper studies on the biological function of solanesol in plants, the medicinal value of solanesol and its derivatives, and the biosynthetic pathway and its key enzymatic genes are of significant medicinal importance.

## 2. Functions of Solanesol

### 2.1. Physiological Functions of Solanesol in Plants

Terpenoids generally function as either primary or secondary metabolites within the plant body. Relatively few terpenoids function as primary metabolites, and these include sterols, carotenoids, and plant hormones, which are necessary for plant growth and physiological functions. For example, sterols composed of triterpenes are important components of the cell membrane that participate in the construction of these membranes; tetraterpenoid carotenoids are pigments that are indispensable for plant photosynthesis, as they participate in the absorption and transfer of light energy and prevent oxidative damage; plant hormones such as gibberellins, abscisic acid, brassinolide, and strigolactones are also terpenoids [8].

Nonetheless, most terpenoids (including solanesol) are secondary metabolites. Various plants produce numerous highly-specific terpenoids that play important roles in plant–environment interactions [3,4]. Solanesol mainly exists in solanaceous plants. In tobacco, solanesol might participate in the immune response towards pathogens: in a study by Bajda et al., the solanesol content in resistant tobacco varieties increased by ≥7 times one week after infection by the tobacco mosaic virus (TMV), while it did not increase significantly after infection in susceptible varieties [7]. In potato, as compared to normal temperatures (22 °C during the day, 16 °C at night), moderately high temperatures (30 °C during the day, 20 °C at night) caused a more than six-fold increase in the solanesol content after one week, indicating that solanesol might play an important role in the response of potato to moderately high temperatures [1]. Hence, solanesol plays important roles in the interactions of solanaceous plants with environmental factors.

### 2.2. Medicinal Value of Solanesol and Its Derivatives

**Figure 1:**
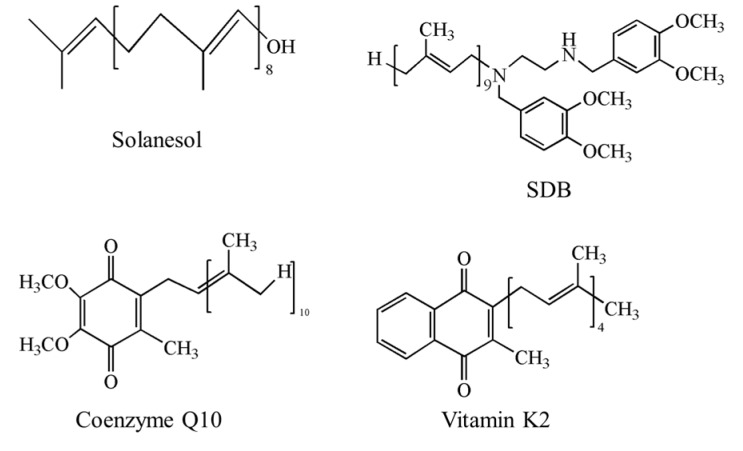
Chemical structures of solanesol and some of its derivatives. SDB: *N*-solanesyl-*N*,*N*′-bis(3,4-dimethoxybenzyl) ethylenediamine.

Solanesol possesses antimicrobial, anti-tumour, anti-inflammatory, and anti-ulcer activities, and it serves as an important pharmaceutical intermediate for the synthesis of coenzyme Q10, vitamin K2, and SDB (Figure 1) [3,6]. The physiological functions of coenzyme Q10 include anti-oxidation, anti-aging, immune-function enhancement, cardiovascular enhancement, brain-function enhancement, and the regulation of blood lipids; it may be used for treating migraines, neurodegenerative diseases, hypertension, and cardiovascular diseases [9,10], and as a dietary supplement for patients with type 2 diabetes [11]. Vitamin K2 promotes bone growth, inhibits bone resorption, stimulates bone mineralization, has preventive and therapeutic effects on osteoporosis, diminishes blood clotting, and reduces the progression of arteriosclerosis [12]. The anti-cancer agent synergizer SDB allows P-glycoprotein-mediated multidrug resistance in cancer cells to be overcome, and has synergistic effects with certain anti-tumour drugs [13,14]. Recently, it was found that solanesol induces the expression of HO-1 and Hsp70, which in turn alleviates alcohol-induced liver cell damage [15]. Additionally, it inhibits the generation of inflammatory cytokines through the p38 and Akt signalling pathways, implying an anti-inflammatory effect [16]. Therefore, solanesol and its derivatives are highly valuable from a pharmaceutical perspective.

## 3. Biosynthetic Pathway of Solanesol

**Figure 2:**
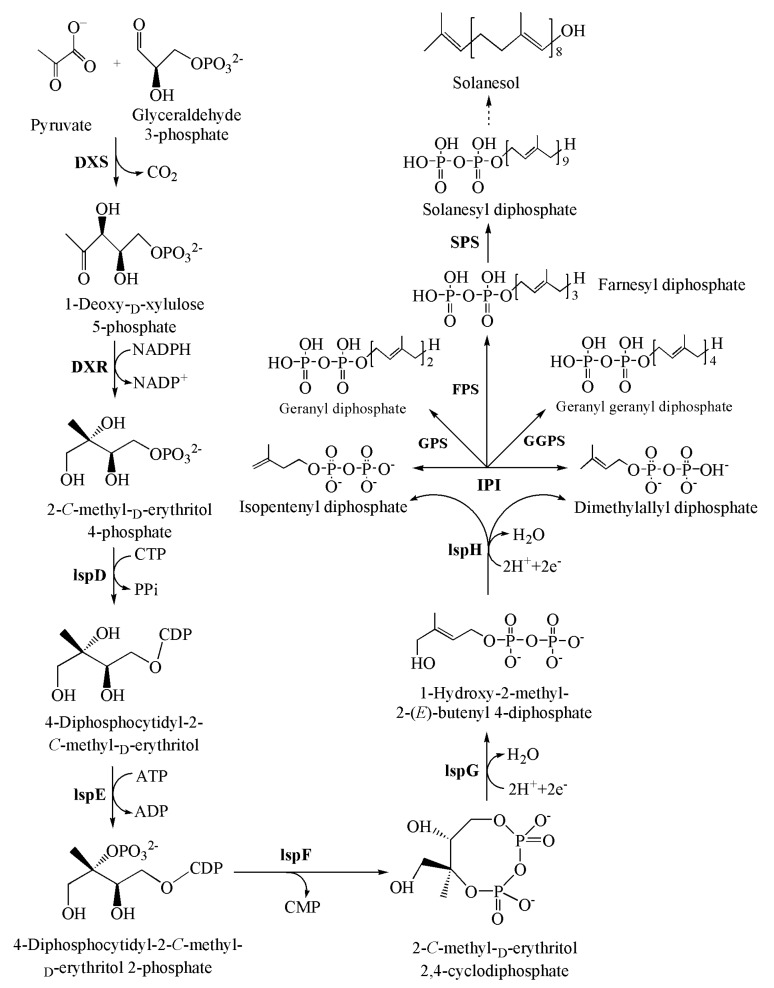
The solanesol biosynthetic pathway in plastids of higher plants. Enzymes involved in the solanesol biosynthetic pathway: DXS, 1-deoxy-d-xylulose 5-phosphate synthase; DXR, 1-deoxy-d-xylulose 5-phosphate reductoisomerase; IspD, 2-*C*-methyl-d-erythritol 4-phosphate cytidylyltransferase; IspE, 4-diphosphocytidyl-2-*C*-methyl-d-erythritol kinase; IspF, 2-*C*-methyl-d-erythritol 2,4-cyclodiphosphate synthase; IspG, 1-hydroxy-2-methyl-2-(*E*)-butenyl 4-diphosphate synthase; IspH, 1-hydroxy-2-methyl-2-(*E*)-butenyl 4-diphosphate reductase; IPI, isopentenyl diphosphate isomerase; GPS, geranyl diphosphate synthase; FPS, farnesyl diphosphate synthase; GGPPS, geranyl geranyl diphosphate synthase; SPS, solanesyl diphosphate synthase.

The biosynthetic pathway of terpenoids generally involves three stages: the generation of the C5 isopentenyl diphosphate (IPP) precursor and its double-bond isomer dimethylallyl diphosphate (DMAPP), the generation of direct precursors (geranyl diphosphate (GPP), farnesyl diphosphate (FPP), geranyl geranyl diphosphate (GGPP), etc.), and the biosynthesis and modification of terpenes via oxidation–reduction, acylation, and glycosylation (Figure 2) [8]. The first two stages of this pathway are well understood; however, because of the relatively simple structure of solanesol, studies on the third stage of biosynthesis remain very scarce.

### 3.1. Formation of C5 Units

IPP and its isomer DMAPP are synthesized via two pathways that occur in different subcellular spaces; i.e., the mevalonate pathway located in the cytoplasm that uses acetyl coenzyme A as a building block, and the 2-*C*-methyl-d-erythritol 4-phosphate (MEP) pathway in plastids that uses pyruvate and glycerol-3-phosphate. These two pathways are not independent of each other, as IPP is shuttled between the cytoplasm and plastids in plants such as tobacco, *Arabidopsis thaliana*, and snapdragon [17,18].

Solanesol is biosynthesized in plastids through the MEP metabolic pathway (Figure 2) [3,6]. 1-deoxy-d-xylulose 5-phosphate synthase (DXS) catalyzes the formation of 1-deoxy-d-xylulose 5-phosphate (DXP) from pyruvate and glycerol-3-phosphate, and DXP undergoes molecular re-arrangement and reduction to form MEP through the catalytic action of 1-deoxy-d-xylulose 5-phosphate reductoisomerase (DXR) (Figure 2). MEP then forms IPP and DMAPP through the successive catalytic actions of 2-*C*-methyl-d-erythritol 4-phosphate cytidylyltransferase (IspD), 4-diphosphocytidyl-2-*C*-methyl-d-erythritol kinase (IspE), 2-*C*-methyl-d-erythritol 2,4-cyclodiphosphate synthase (IspF), 1-hydroxy-2-methyl-2-(*E*)-butenyl 4-diphosphate synthase (IspG), and 1-hydroxy-2-methyl-2-(*E*)-butenyl 4-diphosphate reductase (IspH) (Figure 2). The interconversion between IPP and DMAPP is catalysed by isopentenyl diphosphate isomerase (IPI) [3,6].

### 3.2. Polymerization of C5 Units

The IPP and DMAPP C5 units are catalysed by geranyl diphosphate synthase (GPS), farnesyl diphosphate synthase (FPS), and geranyl geranyl diphosphate synthase (GGPPS) to form GPP, FPP, and GGPP, respectively (Figure 2), which are the precursors of monoterpenes, sesquiterpenes, and diterpenes, respectively [19]. GPS catalyzes the reaction in which one IPP molecule and one DMAPP molecule form a GPP (C10) molecule, while FPS catalyzes the formation of each FPP (C15) molecule from two IPP molecules and one DMAPP molecule through a two-step condensation reaction [20]. GGPPS catalyzes the formation of GGPP (C20) from three IPP molecules and one DMAPP molecule [20]. Solanesyl diphosphate synthase (SPS) catalyzes the formation of solanesyl diphosphate (SPP) by IPP, DMAPP, GPP, FPP, and GGPP (Figure 2) [21,22,23,24], and solanesol is then formed by the conversion of the SPP pyrophosphate groups to hydroxyl groups.

## 4. Key Enzymes in Solanesol Biosynthesis

### 4.1. 1-Deoxy-d-xylulose 5-Phosphate Synthase (DXS)

DXS is the first enzyme of the MEP metabolic pathway that catalyzes the formation of DXP by pyruvate and glycerol-3-phosphate (Figure 2) [3,6]. *DXS* genes have been identified in *A. thaliana* [25], *Medicago truncatula* [26], and *S. lycopersicum* [27,28]. Overexpression or silencing of *DXS* genes in *A. thaliana* significantly altered the contents of terpenoids such as chlorophyll, carotenoids, tocopherols, abscisic acid, and gibberellin [25]. Two *DXS* genes have been identified in *M. truncatula*: *MtDXS1* is expressed in all plant tissues except the roots, while *MtDXS2* is highly expressed only in roots colonized by mycorrhizal fungi [26]. In *S. lycopersicum*, the level of *DXS* gene expression is positively correlated with the carotenoid content in fruits [27]; silencing of the tomato *SlDXS2* gene causes decreases in the β-phellandrene content [28]. Recently, Campbell et al. [1] discovered that transient expression of potato *DXS1* and *DXS2* in *N. benthamiana* significantly increases the solanesol content. Hence, *DXS* is the first key enzymatic gene of the solanesol biosynthetic pathway, and its overexpression or inhibition induces changes in the contents of downstream metabolites.

### 4.2. 1-Deoxy-d-xylulose 5-Phosphate Reductoisomerase (DXR)

DXR catalyzes the molecular rearrangement and reduction of DXP to form MEP (Figure 2) [3,6]. The *DXR* gene has been identified in *Herba menthae* [29], *S. lycopersicum* [30], *Zea mays* [31], *Hevea brasiliensis* [32], and *N. tabacum* [33]. Overexpression of *DXR* genes leads to increases in the chlorophyll and carotenoid contents of *A. thaliana* leaves [34]. Overexpression of *DXR* genes in *H. menthae* increased the production of mint essential oils by 50% [35]. Wu et al. [36] cloned the *DXR* genes of *Salvia miltiorrhiza* and found that their expression increased under high osmotic pressure and fungal elicitor treatment, with expression being positively correlated with the tanshinone content. Zhang et al. [33] identified two *DXR* genes in *N. tabacum*, *NtDXR1* and *NtDXR2*; southern blotting and genotyping indicated that *NtDXR1* and *NtDXR2* originate from *Nicotiana tomentosiformis* and *Nicotiana sylvestris*, respectively. Overexpression of the tobacco *DXR* genes in chloroplasts significantly increased the content of terpenoids, including solanesol [37]. Transient expression of potato *DXR* genes in *N. benthamiana* significantly increased the solanesol content [1]. Hence, *DXR* genes are key to solanesol biosynthesis, and their overexpression promotes the accumulation of downstream metabolites such as solanesol.

### 4.3. Isopentenyl Diphosphate Isomerase (IPI)

IPI catalyzes the conversion of IPP into DMAPP, using Mg^2+^ as a cofactor (Figure 2) [3,6]. *IPI* genes have been identified in *N. tabacum* [38], *A. thaliana* [39,40], *S. lycopersicum* [41], and *Catharanthus roseus* [42]. In *N. tabacum*, two *IPI* genes have been found (i.e., *IPI1* and *IPI2*), which are expressed in the chloroplasts and cytoplasm, respectively [38]. High salinity and irradiation promoted the expression of *IPI1*, while high salinity with low temperature increased the expression of *IPI2*, and treatment with 100 μmol/L exogenous abscisic acid increased the expression of both genes [38]. Two *IPI* genes have been identified in *A. thaliana*: *AtIPI1* and *AtIPI2*, which are expressed in the plastids and mitochondria, respectively [39]. *AtIPI1* or *AtIPI2* single mutants appear to be normal, while double mutant plants display dwarfing and male sterility, and sterol and ubiquinone contents that are decreased by over 50% as compared to the wild type [40]. *Escherichia coli* expressing the yeast *Saccharomyces cerevisiae* *IPI* genes produces a variety of terpenoids [43]. Introduction of *IPI* genes into single-cell green algae dose-dependently increased their carotenoid content [44]. Co-expression of potato *IPI* and *SPS* genes in *N. benthamiana* significantly increased the solanesol content [1]. Hence, *IPI* is a key enzymatic gene in the biosynthesis of terpenoids, and its overexpression or inhibition induces changes in the contents of downstream metabolites.

### 4.4. Geranyl Geranyl Diphosphate Synthase (GGPPS)

*GGPPS* catalyzes the condensation of three IPP molecules with one DMAPP molecule to form one GGPP molecule (Figure 2) [19]. GGPP is a common precursor in the synthesis of diterpenes, tetraterpenes, and polyterpenes, and it participates in the synthesis of chlorophyll, carotenoids, cytokinins, gibberellins, abscisic acid, plastoquinone, ubiquinone, and solanesol [45,46]. Plant GGPPSs may be divided into large or small subunits; nine members of the GGPPS family have been identified in *N. tabacum*, seven of which are large subunits and two of which are small subunits; five members of the GGPPS family have been identified in *N. tomentosiformis* and *N. sylvestris*, among which four are large subunits and one is a small subunit [47]. NtGGPPS3 is a large subunit located in chloroplasts and the plasma membrane, and the *NtGGPPS3* gene is expressed in all tobacco tissues during all major stages of growth, with especially high levels in leaves and stems [48,49]. NtGGPPS5 is a small subunit, and *NtGGPPS5* is expressed in the roots, stem, leaves, and buds of tobacco, with expression decreasing in the order: bud > leaf > stem > root [50]. After treatment with methyl jasmonate, the expression of *NtGGPPS1* increased significantly, whereas its expression decreased following auxin treatment, and silencing of *NtGGPPS1* significantly lowered the levels of chlorophyll and carotenoid [51]. In the report by Campbell et al. [1], transient expression of the potato *GGPPS3* gene in *N. benthamiana* significantly increased solanesol content. Hence, *GGPPS* is a key enzymatic gene in terpenoid biosynthesis, and its overexpression promotes the accumulation of downstream metabolites such as solanesol.

### 4.5. Solanesyl Diphosphate Synthase (SPS)

SPS catalyzes the synthesis of SPP from IPP, DMAPP, GPP, FPP, and GPPP, and SPP is a precursor in solanesol and plastoquinone synthesis (Figure 2) [3,6]. To date, *SPS* genes have been identified in *A. thaliana* [21,22,52], *H. brasiliensis* [53], *Oryza sativa* [23], *S. lycopersicum* [24], and *N. tabacum* [6]. *A. thaliana* carries two *SPS* genes: *AtSPS1* and *AtSPS2*, the expression of which is significantly higher in leaves and stems than in roots [22]. Silencing of *AtSPS1* and *AtSPS2* lowered the leaf plastoquinone content, thus inducing photoinhibition [52]. Fibrillin 5 (FBN5) binds with AtSPS1 and AtSPS2 to regulate the synthesis of plastoquinone [54]. In *O. sativa*, two *SPS* genes have been identified: *OsSPS1* and *OsSPS2*, which are expressed in the mitochondria and plastids, respectively; OsSPS1 preferentially catalyzes the synthesis of ubiquinone-9 from FPP in mitochondria, while OsSPS2 preferentially catalyzes the synthesis of plastoquinone-9 from GPP in plastids [23]. In tobacco, *NtSPS1* and *NtSPS2* are expressed in various organs, with the levels decreasing in the order leaf > stem > root, which is consistent with the distribution of solanesol and chlorophyll in these organs [55]. NtSPS1 and NtSPS2 are both located in chloroplasts, which is consistent with the subcellular localization of *S. lycopersicum* SPS [24]. NtSPS1 and NtSPS2 both contain two conserved DDxxD domains that participate in coordinating the binding between divalent metal ions and pyrophosphate groups, and thus serve an important role in the positioning of the reaction substrates [8]. Overexpression of tomato *SPS* in tobacco significantly increased the plastoquinone content of immature leaves and the solanesol content of mature leaves [24], and co-expression of potato *SPS* genes with *DXS*, *DXR*, *IPI*, and *GGPPS3* significantly increased the solanesol content of *N. benthamiana* [1]. Hence, *SPS* is a key enzymatic gene for the biosynthesis of solanesol, and its overexpression promotes the accumulation of downstream metabolites such as solanesol.

## 5. Conclusions and Perspectives

Solanesol is a non-cyclic terpene alcohol that consists of nine isoprene units and mainly accumulates in solanaceous plants such as tobacco, potato, and tomato. Solanesol serves an important role in the interactions between plants and their environment, and it is a key intermediate for the pharmaceutical synthesis of ubiquinone-based supplements and drugs. Notably, although solanesol and its derivatives are highly valuable from a pharmaceutical perspective, solanesol as a C45 compound may act as a tumorigenic precursor in tobacco smoke [56,57]. While in recent years, studies on the identification of key enzymatic genes in solanesol biosynthesis and gene function have achieved significant progress, a number of questions on the regulatory mechanisms of solanesol synthesis remain unanswered. Genome sequencing of solanaceous plants such as tobacco [58], potato [59], and tomato [60] has paved the way for deeper studies on the metabolic regulation of solanesol biosynthesis. Transcriptomics and metabolomics studies may aid in resolving the metabolic flux distribution of solanesol and the mechanisms through which it interacts with other metabolic pathways. From the metabolomics point of view, it would be interesting to analyse which metabolites are secondarily produced after disruption of some of the solanesol biosynthetic genes. We have generated *NtSPS1*-overexpressing tobacco plants in our laboratory, which will allow us to evaluate the effects of *SPS1* overexpression on solanesol and related metabolites, photosynthesis, and the expression levels of key solanesol biosynthetic and related genes in tobacco. Moreover, overexpression of key enzymatic genes will allow tobacco plants with high solanesol content to be obtained, with significant importance for medical applications. Microbial heterologous expression of key tobacco enzymatic genes may be used to identify their function and to generate solanesol derivatives of medicinal value.
